# Cystatin C Mirrors Fibrosis Burden in Metabolic Syndrome: Insights from the Metabolic Dysfunction-Associated Fibrosis-5 Score

**DOI:** 10.3390/metabo16010071

**Published:** 2026-01-13

**Authors:** Musa Salmanoğlu, Sinan Kazan, Elif Yıldırım Ayaz, Süleyman Kılıç, Elif Kazan, Sena Ulu

**Affiliations:** 1 Department of Internal Medicine, Sultan 2. Abdülhamid Han Training and Research Hospital, University of Health Sciences, Selimiye, Tıbbiye Cd, 34668 İstanbul, Turkey; drmusa1973@gmail.com (M.S.); silimoney@gmail.com (S.K.);; 2 Department of Nephrology, Afyonkarahisar Health Sciences University, Battalgazi, Ahmet Necdet Sezer Kampüsü, 03204 Afyonkarahisar, Turkey; sinankazan@hotmail.com; 3 Department of Internal Medicine, Afyonkarahisar Health Sciences University, Battalgazi, Ahmet Necdet Sezer Kampüsü, 03204 Afyonkarahisar, Turkey; elif.kazan@afsu.edu.tr

**Keywords:** cystatin C, MAF-5, metabolic syndrome, hepatic fybrosis

## Abstract

Background: Metabolic syndrome (MetS) comprises interrelated metabolic abnormalities that collectively confer increased risk of cardiovascular disease and hepatic morbidity. The MAF-5 score is a non-invasive prognostic marker of liver fibrosis and mortality, while Cystatin C (CysC) is a sensitive indicator of renal function that also reflects inflammation, atherosclerosis, and metabolic dysfunction. Although both MetS and CysC have been widely studied, their potential interplay via MAF-5 remains unclear. We aimed to investigate the relationship between MAF-5 scores and CysC levels in MetS patients for the first time. Materials and Methods: Adults (≥18 years) with MetS were included in this study. MAF-5 scores (based on waist circumference, BMI, diabetes status, AST, and platelet count) and CysC levels were recorded. The MAF-5–CysC relationship was assessed via Pearson correlation. Participants were grouped into MAF-5 quartiles, and continuous variables were compared using ANOVA with Bonferroni-corrected pairwise tests. Results: We included 347 MetS patients (54.8% female, median age 54 years). The median MAF-5 score was 1.25, and MAF-5 correlated positively with CysC (r = 0.357, *p* < 0.001). CysC levels differed significantly across MAF-5 quartiles (Q1 = 0.96, Q2 = 0.99, Q3 = 1.06, Q4 = 1.09; *p* < 0.001), with Q4 showing higher values than Q1 and Q2. Conclusions: A significant correlation was found between MAF-5 scores and CysC in patients with MetS. CysC levels differed significantly across MAF-5 quartiles, suggesting a potential link between systemic inflammation, liver fibrosis, and CysC. These results highlight shared inflammatory and fibrotic pathways, underlying metabolic dysfunction. Clinically, combined assessment of MAF-5 and CysC may improve risk stratification, identifying patients at higher risk for hepatic fibrosis and adverse outcomes.

## 1. Introduction

Metabolic syndrome (MetS) is a complex clinical entity characterized by the coexistence of cardiometabolic risk factors including insulin resistance, abdominal obesity, dyslipidemia, and hypertension [1,2,3,4]. MetS frequently coexists with metabolic dysfunction-associated steatotic liver disease (MASLD), and the prevalence of both conditions has been steadily increasing in parallel with rising obesity rates and lifestyle-related factors [2,5,6]. Current evidence indicates that MASLD represents not only a hepatic disorder but also a manifestation of systemic metabolic dysfunction [7]. With the advancement of therapeutic strategies, the prevention and early identification of MASLD have become key therapeutic targets in the management of metabolic syndrome [8].

Recently, the Metabolic Dysfunction-Associated Fatty Liver Disease Risk Score 5 (MAF-5) has been introduced as a novel noninvasive scoring system aimed at the early detection of MASLD and the identification of individuals at high risk, demonstrating high accuracy and reliability in predicting liver stiffness and fibrosis risk [9].

MASLD is commonly associated with inflammation and fibrotic processes. During these processes, the expression of various human cystatins increases. Among them; Cystatin C (CysC) is one of the best-characterized endogenous cysteine protease inhibitors [10]. CysC, classically recognized as a renal function biomarker, but it has recently been reported to be elevated in various fibrotic conditions, including hepatic, cardiac, renal, and pulmonary fibrosis, where its upregulation correlates closely with disease severity, tissue remodeling [10,11]. In the literature, Cystatin C has been shown to be significantly associated with coronary atherosclerosis, likely due to its involvement in both inflammatory and fibrotic pathways [12,13]. These findings suggest that CysC is an active participant in the fibrogenic process, supporting its potential role as a noninvasive biomarker reflecting fibrotic activity in MASLD.

To the best of our knowledge, no previous studies have investigated the relationship between MAF-5 score, used to identify hepatic injury, and CysC levels in patients with MetS.

The present study aimed to investigate the relationship between the MAF-5 score and serum CysC levels. Given that both markers are closely linked to metabolic dysfunction, systemic inflammation, and fibrotic pathways, it is plausible that CysC may reflect the extent of hepatic injury and fibrosis indicated by the MAF-5 score. We hypothesized that individuals with higher MAF-5 scores would exhibit significantly elevated serum CysC levels, supporting the potential role of CysC as a surrogate biomarker indicative of fibrotic progression in MASLD.

## 2. Material and Methods

### 2.1. Study Design and Setting

This cross-sectional study was conducted at Sultan Abdulhamid Han Training and Research Hospital between 1 April 2024, and 31 August 2025. The study protocol was systematically reviewed and subsequently approved by the University of Health Sciences Hamidiye Ethics Committee. Written informed consent was obtained from all participants prior to their enrollment, in strict accordance with the principles outlined in the Declaration of Helsinki and international ethical guidelines for human research. Participants were recruited from adult individuals presenting to the hospital’s outpatient clinics for routine control. Inclusion and exclusion criteria were carefully defined to ensure a representative sample of the general adult population while maintaining participant safety and data integrity. All clinical assessments, laboratory analyses, and study procedures were performed under standardized conditions, with rigorous adherence to institutional ethical standards. Participant confidentiality was ensured, and study procedures were conducted to minimize bias.

**Figure 1:**
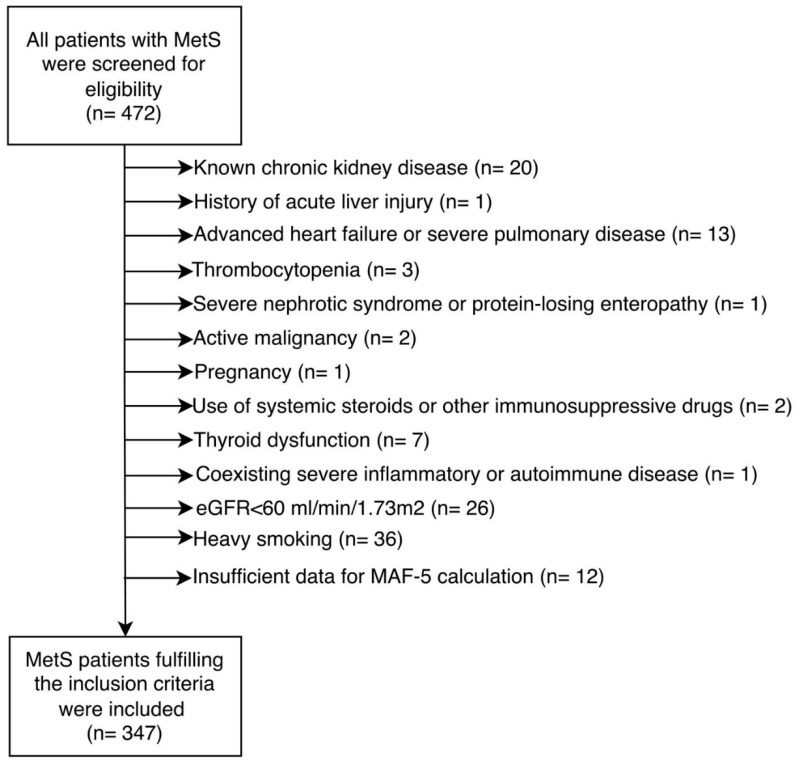
The exclusion criteria are presented in the flowchart.

All patients aged 18 years and older who were followed up with a diagnosis of MetS were screened for eligibility. The diagnosis of MetS was established according to the criteria of the National Cholesterol Education Program Adult Treatment Panel III (NCEP ATP III) [1]. Accordingly, the presence of at least three of the following five components was required to confirm the diagnosis: increased waist circumference (≥102 cm in men or ≥88 cm in women), elevated blood pressure (≥130/85 mmHg) or current use of antihypertensive medication, fasting triglyceride level of ≥150 mg/dL, reduced high-density lipoprotein cholesterol (HDL-C) (<40 mg/dL in men or <50 mg/dL in women), and fasting plasma glucose level of ≥100 mg/dL or current use of antidiabetic medication. To minimize potential confounding factors that could influence serum CysC levels or the MAF-5 score, several exclusion criteria were applied. Patients with known chronic kidney disease, a history of acute liver injury, advanced heart failure, or severe pulmonary disease were excluded. Individuals with thrombocytopenia, severe nephrotic syndrome, or protein-losing enteropathy were also not included. Additional exclusion criteria comprised active malignancy, pregnancy, thyroid dysfunction, or the use of systemic corticosteroids and other immunosuppressive agents. Patients with severe inflammatory or autoimmune disorders, an estimated glomerular filtration rate (eGFR) below 60 mL/min/1.73 m^2^, heavy smoking history (>10 pack-years), or insufficient clinical or laboratory data required for MAF-5 score calculation were also excluded from the study. The exclusion criteries are illustrated in Figure 1.

### 2.2. Calculation of MAF-5 Score

MAF-5 score was calculated for each participant using the formula proposed by Van Kleef et al. [14]. The equation is as formula: “−11.3674 + (0.0282 × WC) − (0.1761 × BMI) + (0.0019 × WC × BMI) + (2.0762 × Diabetes) + (2.9207 × ln log (AST)) − (0.0059 × Platelet)”, where WC is waist circumference (cm), BMI is body mass index (kg/m^2^), AST is serum aspartate aminotransferase level (U/L, log-transformed), Platelet denotes platelet count (×10^9^/L), and Diabetes represents diabetes status (coded as 1 for presence and 0 for absence). Higher MAF-5 values indicate a greater probability of hepatic fibrosis and an increased risk of adverse metabolic outcomes. Based on prior validation studies, a score <0 is interpreted as low risk, 0 ≤ score < 1 as intermediate risk, and ≥1 as high risk for significant hepatic fibrosis [14].

### 2.3. Cystatin C Measurement Method

Serum Cystatin C levels, a sensitive biomarker of renal function, were measured using the Siemens BN II Nephelometer (Siemens Healthcare Diagnostics, Marburg, Germany). The measurement was based on an immunonephelometric principle. Calibration of the device was performed according to the manufacturer’s guidelines, and analyses were conducted in accordance with reproducibility and accuracy criteria. The reference range for healthy adult individuals was defined as 0.61–0.95 mg/L. All measurements were performed following standard laboratory operating procedures, and internal quality controls were applied to ensure the reliability of the results.

### 2.4. Statistical Analysis

The association between waist circumference and serum Cystatin C levels was previously reported in elderly individuals, with a correlation coefficient of r = 0.520 [15]. Based on this finding, a power analysis was conducted using G*Power 3.1 software with an assumed effect size of r = 0.25, a significance level of α = 0.05, and statistical power of 0.80, which indicated that a minimum of *n* = 123 participants would be required. With a sample size of *n* = 347, our study achieved over 99% power to detect correlations of r = 0.357 or higher using a two-tailed Pearson test.

Categorical variables are presented as percentages and absolute frequencies. For continuous variables, the normality of distribution was assessed using the Shapiro–Wilk test and visual inspection of histograms. Variables with normal distribution are expressed as mean values with standard deviations, whereas non-normally distributed variables are reported as medians with interquartile ranges (Q1–Q3). The Pearson correlation test was used to investigate the relationship between MAF-5 score and CysC. To compare categorical variables across MAF-5 quartile groups, the chi-square test was employed. For continuous variables, comparisons between groups were made using the analysis of variance (ANOVA) when the data followed a normal distribution, and the Kruskal–Wallis test when the distribution was non-normal. Post hoc analysis of CysC levels among MAF-5 quartile groups was performed using pairwise comparisons test and Bonferroni correction. All statistical analyses were performed using the SPSS software package, version 27.0.1. Two-sided *p*-values were reported, and a *p*-value less than 0.05 was considered indicative of statistical significance.

## 3. Results

**Figure 2:**
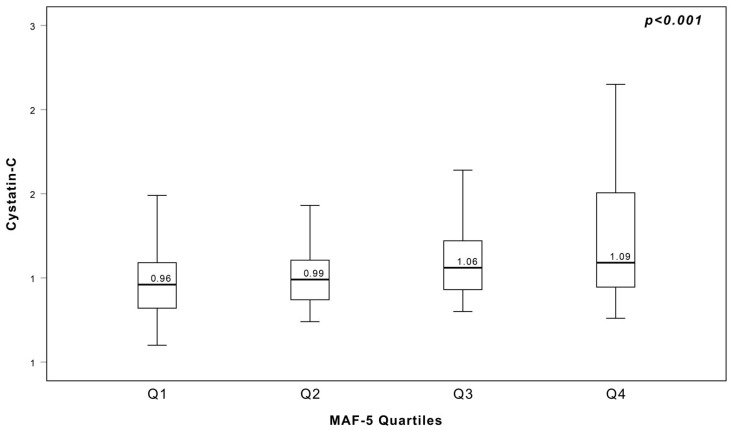
Correlation between MAF-5 score and CysC.

A total of 347 patients with a diagnosis of MetS were enrolled in the study. Of these, 54.8% (*n* = 190) were female and 45.2% (*n* = 157) were male. The overall median age of the study cohort was 54 years (interquartile range [IQR]: 47–60 years). The median MAF-5 score was 1.2499. Correlation analysis revealed a statistically significant positive association between MAF-5 and serum CysC levels (*r* = 0.357, *p* < 0.001). This relationship is graphically illustrated in Figure 2. To further explore this association, patients were stratified into quartiles according to their MAF-5 scores. The first quartile (Q1), defined as a score below −0.1133, comprised 24.8% of the patients (*n* = 86). The second quartile (Q2), ranging between −0.1133 and 1.2498, included 25.1% (*n* = 87). The third quartile (Q3), with scores from 1.2499 to 2.4122, accounted for 24.8% of patients (*n* = 86). The fourth quartile (Q4), defined as a score above 2.4122, represented 25.4% of the study population (*n* = 88). A detailed comparison of demographic, clinical, and biochemical characteristics across the MAF-5 quartiles is summarized in Table 1.

**Figure 3:**
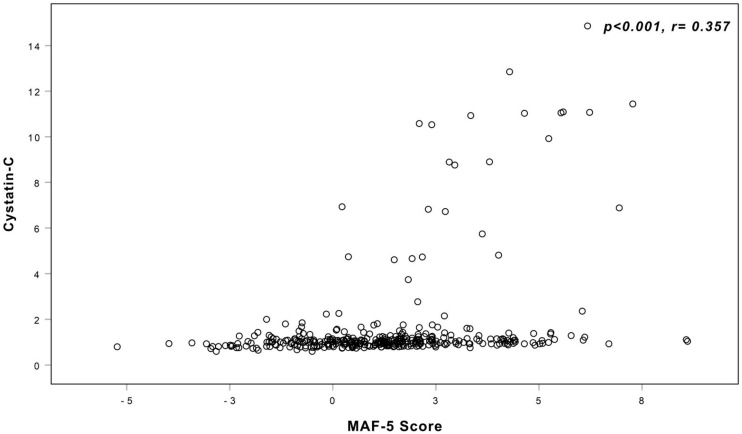
Comparison of CysC across the MAF-5 quartile groups.

A significant difference in CysC levels was observed across the four MAF-5 quartiles (*p* < 0.001), as depicted in Figure 3. Post hoc pairwise analyses demonstrated that this significance was primarily driven by higher CysC concentrations in patients belonging to the Q4 group compared with those in Q1 and Q2. The results of these post hoc comparisons are presented in Table 2.

## 4. Discussion

In this study, we demonstrated, for the first time in the literature, a significant positive association between MAF-5 and serum CysC levels. These results show a link between MAF-5 score (a liver-specific fibrosis index) and CysC (a biomarker that integrates renal, vascular, and inflammatory injury), thereby providing a more holistic view of disease burden in metabolic dysfunction [9]. Previously, the predictive performance of MAF-5 in liver fibrosis, liver-related outcomes, and mortality was established. Our findings demostrate for the first time, an association between MAF-5 and serum CysC levels. This novel observation suggests that the two biomarkers may jointly capture shared biological pathways underlying metabolic dysregulation, systemic inflammation, and fibrogenesis, and may therefore have complementary value in risk stratification.

Yang et al. demonstrated that higher serum CysC levels were significantly associated with the development of MetS after following adults who underwent physical examinations over a two-year period [16]. In a study conducted in the United States among individuals with MetS, CysC was reported to predict both all-cause and cardiovascular mortality [17]. Although several studies have indicated that serum CysC is related to the inflammatory burden observed in MetS, its association with hepatic steatosis—one of the most clinically relevant comorbidities in recent years—has not been adequately investigated.

CysC has increasingly been recognized as a biomarker of systemic inflammation. The Heart and Soul study, demonstrated that serum CysC levels were significantly correlated with established markers of inflammation, including C-reactive protein (CRP) and fibrinogen [18]. A recent prospective cohort and Mendelian randomization study in acute ischemic stroke further demonstrated that elevated CysC levels independently predicted poorer 90-day functional outcomes and increased mortality [19]. These associations were partially mediated by systemic inflammatory burden and supported a potential causal role of CysC in the progression of cerebrovascular disease [19]. These observations add weight to the hypothesis that CysC, although long regarded primarily as a marker of renal function, may also serve as a sensitive surrogate of systemic inflammatory activity and vascular injury. Consistent with this, CysC has been linked to recurrent cardiovascular events and adverse outcomes independent of traditional renal indices, suggesting that it captures a broader cardio-renal–inflammatory phenotype.

**Figure 4:**
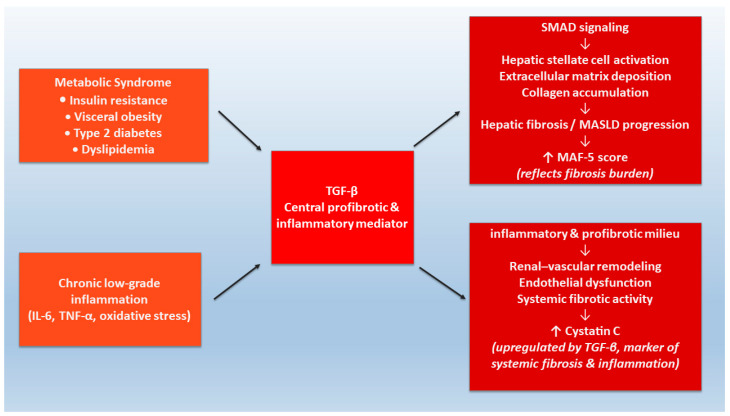
TGF-β–mediated inflammatory and fibrotic pathways underlying the association between MAF-5 score and serum cystatin C.

Transforming growth factor-beta (TGF-β) serve as a central mediator of both inflammatory and fibrotic signaling cascades thereby emerging as a central mechanistic link between these interconnected pathological processes [20,21]. Biological interactions between CysC and TGF-β have been reported in the literature. For example, Sokol et al. demonstrated that TGF-β upregulates CysC transcript and protein expression, while CysC, conversely, can physically interact with TGF-β receptor type II to antagonize TGF-β signaling. Furthermore, mechanistic data support the role of CysC as an extracellular antagonist of TGF-β activity [22]. These findings provide a possible mechanism through which CysC may modulate profibrotic pathways in metabolically injured organs, including the liver, kidney and vasculature (Figure 4).

The MAF-5 score, developed and validated by van Kleef et al., has been shown to outperform traditional non-invasive indices in predicting the risk of liver fibrosis. In addition, elevated MAF-5 values have been associated with increased all-cause mortality. More recently, Korula et al. reported that in adults with metabolic dysfunction, the MAF-5 score effectively predicted fibrotic risk [21]. Although the MAF-5 score is a powerful index for predicting liver fibrosis, it is calculated using parameters such as age, BMI, AST/ALT ratio, triglyceride level, and the presence of type 2 diabetes—all of which reflect systemic inflammatory and fibrotic burden. In this context, our finding of a significant correlation between MAF-5 and serum CysC suggests that both markers are overlapping pathophysiological processes. While MAF-5 primarily represents hepatic and metabolic fibrogenesis [9], CysC emerges as a parallel indicator of systemic inflammation and extracellular matrix remodeling [10], thereby establishing a biological bridge between hepatic and renal–vascular axes within the spectrum of metabolic dysfunction. Notably, Song et al. identified CysC as the strongest predictor of both all-cause and cardiovascular mortality in patients with MetS, demonstrating superior prognostic performance compared to eGFR, blood urea nitrogen, creatinine, uric acid, and CRP [17]. This observation is noteworthy, as the findings of our study focus specifically on the MAFLD–CysC relationship and underscore the growing importance of CysC as a biomarker extending beyond renal fibrosis. Taken together, these data suggest that integrating MAF-5 and CysC may allow clinicians to capture both organ-specific (hepatic) fibrosis and systemic cardio-renal–metabolic risk within a single, easily obtainable biomarker framework [23].

From a clinical standpoint, the implications of these findings are considerable. Assesment of MAF-5 and CysC together may provide clinicians a more comprehensive, multidimensional understanding of the disease burden in MetS. Patients who simultaneously exhibit high MAF-5 scores and elevated CysC levels may constitute a particularly vulnerable subgroup at increased risk not only for progressive hepatic fibrosis but also for accelerated cardiometabolic deterioration and heightened mortality. In this high-risk phenotype, early referral for elastography, close cardiometabolic follow up, and optimization of lifestyle and pharmacologic therapy (including glucose-, lipid- and blood pressure-lowering interventions with proven organ-protective effects) may be justified [9,24].

This study has several limitations. First, the cross-sectional design precludes the establishment of a causal relationship between serum CysC levels and the MAF-5 score; therefore, the observed association should be interpreted as correlative rather than causal. Second, although the sample size was statistically adequate, all participants were recruited from a single tertiary care center, which may limit the generalizability and external validity of the findings.

## 5. Conclusions

In conclusion, this study is the first in the literature to investigate the relationship between the MAF-5 score and serum CysC levels, offering a novel perspective on the shared pathophysiological mechanisms linking hepatic and systemic fibrogenesis. The integration of MAF-5 and CysC—both low-cost and readily accessible biomarkers—into clinical practice may represent a meaningful advancement in the management of patients with metabolic syndrome (MetS), enabling earlier identification of individuals at increased risk. Future studies should assess whether MAF-5–CysC-based algorithms provide incremental prognostic value over existing risk scores and whether biomarker-guided interventions can modify hard outcomes such as liver-related events, cardiovascular events, and mortality.

## Figures and Tables

**Table 1:** General characteristics of all patients and comparison of MAF-5 quartile groups.

Parameters	All Patients	Q1 (*n* = 86)	Q2 (*n* = 87)	Q3 (*n* = 86)	Q4 (*n* = 88)	*p*
Gender, female (%-*n*)	54.8–190	58.1–50	56.3–49	51.2–44	53.4–47	0.802
Age (years)	54 (47–60)	52.5 (44–61)	55 (48–60)	57 (47.8–61)	53 (47–61)	0.884
DM (%-*n*)	76.1–264	45.3–39	81.6–71	90.7–78	86.4–76	<0.001
HT (%-*n*)	52.4–182	44.2–38	47.1–41	60.5–52	58–51	0.085
HL (%-*n*)	49.9–173	32.9–28	50.6–44	61.6–53	54.5–48	0.002
BMI (kg/m^2^)	31.5 (27.9–36.1)	28.4 (25.7–31.5)	29.9 (26.9–33.2)	32.8 (29.9–36.5)	36.2 (32.7–41.9)	<0.001
Waist circumference (cm)	108.2 ± 13.7	97.53 ± 9.5	103.35 ± 8.5	109.56 ± 8.8	121.7 ± 14.6	<0.001
Smoking (%-*n*)	26.2–91	32.6–28	26.4–23	20.9–18	25–22	0.377
Alcohol (%-*n*)	10.1–35	9.3–8	13.8–12	10.5–9	6.8–6	0.489
Systolic BP (mmHg)	144 (130–158)	140 (124–153.5)	143 (132.5–156.5)	144.5 (130–158)	145 (134–163)	0.003
Diastolic BP (mmHg)	86 (79–94)	85 (77.5–93)	85 (78.5–92)	85.5 (80–96)	86 (79–97)	0.306
FPG (mg/dL)	125 (102–171)	109 (94–160)	126 (109–176)	136 (109–181)	126 (105–161)	0.040
HbA1c (%)	6.95 (6.1–8.8)	6.2 (5.73–8.4)	6.7 (6.1–8.85)	7.7 (6.4–8.8)	7.05 (6.2–8.9)	0.002
TC (mg/dL)	192.2 ± 46.3	204.32 ± 47.9	186.27 ± 42.2	184.94 ± 46.9	185.27 ± 43.6	0.049
Triglycerides (mg/dL)	137.2 (97–214)	122 (78–178)	134 (92–206)	157 (111–219)	147 (115–240)	<0.001
HDL-C (mg/dL)	45 (38–53)	50 (41–56)	45 (40–55)	43 (38–52)	43 (37–50)	0.004
LDL-C (mg/dL)	109 (83–137)	124 (98–150)	107 (75–137)	104 (81–130)	103 (80–125)	<0.001
WBC (×10^3^/μL)	7.61 (6.5–8.99)	7.38 (6.41–9.07)	7.51 (6.15–8.56)	7.67 (6.6–8.74)	7.83 (6.82–9.28)	0.258
Hb (g/dL)	13.85 ± 1.6	13.59 ± 1.6	13.9 ± 1.5	13.87 ± 1.5	13.89 ± 1.8	0.200
PLT (×10^3^/μL)	260 (217–311)	283 (232–334)	250 (221–311)	268 (216–304)	250 (199–290)	<0.001
Creatinine (mg/dL)	0.8 (0.7–0.94)	0.83 (0.69–0.95)	0.82 (0.71–0.95)	0.84 (0.73–0.94)	0.77 (0.70–0.89)	0.282
eGFR (mL/min/1.73 m^2^)	93.8 (82–105)	93.7 (77.8–105.1)	95.5 (80.9–103.6)	90.5 (79.6–100.9)	96.6 (84.5–106.6)	0.195
AST (U/L)	36 (28.5–37)	36.3 (33.3–37)	36.3 (33.1–37)	35 (23.6–37)	34.7 (26.4–36.9)	0.004
ALT (U/L)	19.8 (14.3–29.5)	14.5 (11.9–18.6)	18.9 (13.7–26.7)	20.5 (16.7–28.1)	30.7 (20.7–44.3)	<0.001

DM = diabetes mellitus, HT = hypertension, HL = hyperlipidemia, BMI = body mass index, BP = blood pressure, FPG = fasting blood glucose, TC = total cholesterol, HDL-C = high-density lipoprotein cholesterol, LDL-C = low-density lipoprotein cholesterol, WBC = White blood cell, Hb = hemoglobin, PLT = platelets, eGFR = estimated-Glomerular Filtration Rate, AST = aspartate aminotransferase, ALT = alanine aminotranferasee.

**Table 2:** Post hoc analysis of MAF-5 groups in terms of CysC levels.

MAF-5 Groups	Adjusted Significance *
Q1–Q2	1.000
Q1–Q3	0.057
Q1–Q4	0.009
Q2–Q3	0.345
Q2–Q4	0.048
Q3–Q4	1.000

* *p*-values have been adjusted by the Bonferroni correction.

## Data Availability

All datasets on which the results of an article were based were included as part of the submission. The datasets generated or analyzed during this study are available from the corresponding author on reasonable request.
